# Vocal fold leukoplakia recurrence risk model

**DOI:** 10.1038/s41598-023-50691-3

**Published:** 2024-01-02

**Authors:** Hanna Klimza, Joanna Jackowska, Wioletta Pietruszewska, Andrzej Porębski, Piotr Nogal, Agata Leduchowska, Małgorzata Wierzbicka

**Affiliations:** 1 Regional Specialist Hospital Wroclaw, Research & Development Centre, Kamieńskiego 73a, 51-124 Wrocław, Poland; 2 Wroclaw University of Science and Technology, 27 Wybrzeże Stanisława Wyspiańskiego St, 50-370 Wroclaw, Poland; 3 Institute of Human Genetics Polish Academy of Sciences, Strzeszynska 32, 60-479 Poznań, Poland; 4 Department of Otolaryngology, Head and Neck Surgery, Poznań University of Medical Sciences, Przybyszewskiego Street 49, 60-355 Poznań, Poland; 5 Department of Otiatrics, Laryngology and Laryngological Oncology, Medical University of Łódź, Kościuszki Alley 4, 90-419 Łódź, Poland; 6 Faculty of Law and Administration, Jagiellonian University, 24 Gołębia St., 31-007 Kraków, Poland

**Keywords:** Imaging techniques, Cancer prevention

## Abstract

The paper aims to define the variables that elevate the risk of VFL recurrence after adequate primary treatment, and to present the Recurrence Risk Model with practical conclusions to handle pVFL and rVFL. Out of 207 patients with primary vocal fold leukoplakia (pVFL), in 41 (19.8%) recurrent VFL (rVFL) was diagnosed. All patients were assessed by using a trans-nasal flexible video-endoscope using white light, and NBI. The primary measure of our study was to investigate whether morphological features of pVFL in WL, vascular pattern in NBI, and primary histological findings could predict VFL recurrence. To create a model of risk factors, two methods were used: logistic regression and a conditional inference decision tree. The study showed smoking was the factor that significantly and most strongly increased the likelihood of rVFL, as well as the older age groups have a greater odds of rVFL. Types IV, V and VI, according to Ni 2019 classification, were associated with a significantly higher risk of rVFL. The algorithm combining patients’ dependent variables and the combination of two classifications improves the predictive value of the presented VFL Recurrence Risk Model.

## Introduction

Vocal fold leukoplakia (VFL) is a medical condition characterized by the development of white patches or plaques on the surface of the VF. The condition is considered recurrent, if the white patches reappear after adequate treatment. Plaques contain varying amounts of keratin and are histologically determined from hyperkeratosis, through all degrees of dysplasia to carcinoma in situ or even invasive cancer^1^. The prevalence of VFL is estimated at 10.2 for men and 2.1 for women per 100,000 persons^2^. The risk of malignant transformation ranged from 1 to 40%^1,3^.

VFL is still a controversial topic in terms of diagnostics and management; however, the majority of practitioners recommend surgical treatment for all patients with this condition^4–6^. The philosophy of VFL surgery is to maintain a balance between removing the lesion and preserving voice. Some authors presented an algorithm to categorize patients into those referred to watchful waiting, dedicated to strictly selected cases, while others were referred for surgery without undue delay^7^. Unfortunately, in some patients, recurrence of VFL (rVFL), despite appropriate treatment, can be observed. In the most popular therapeutic option, i.e. transoral laser microsurgery with carbon dioxide laser (TOLM CO2), the recurrence rate ranged from 16 to 22%^8,9^. Repeated procedures can be connected with deteriorating the mucosa and poorer voice outcomes, but hard data on this subject is lacking. What is more, in up-to-date literature, there are no defined clear risk factors of VFL recurrence and procedures to handle rVFL, if the condition is mild. Thus, we hypothesize that the causes of rVFL have been still unclear and poorly documented in the literature, therefore it would be necessary to retrospectively analyze patients treated for primary VFL (pVFL) to identify factors that increase the risk of relapse.

The paper aims to define the variables that elevate the risk of VFL recurrence after adequate primary treatment, and to present the Recurrence Risk Model with practical conclusions to handle pVFL and rVFL. The “adequate” treatment means complete excision of the vocal fold leukoplakia. Based on the output data, we want to define the conditions in which more intensive pVFL treatment should have been advised.

## Material and methods

The Ethic Committee of the Medical University of Łódź approved the study, including any relevant details. All experiments were performed in accordance with relevant named guidelines and regulations. Informed consent was obtained from all participants and/or their legal guardians.

A retrospective study was carried out at the Poznan and Łódź Otolaryngology Tertiary Referral University Departments, between January, 2015 and March, 2020 (decision no. RNN/225/19/KE, 9th April, 2019).

For this study, 207 consecutive patients with pVFL, with a total number of 255 lesions, were enrolled. Out of 207 patients, 41 (19.8%) rVFL was diagnosed. In the control group, not including cases identified as cancer, there were 166 patients without recurrence.

Inclusion criteria for the rVFL group: The patients (I) > 18 years of age, (II) with rVFL (IIIa) who required ≥ 2 direct laryngeal surgery (IIIb) with a minimum of 6-month interval between each procedure. An adequate first treatment with complete VFL excision was administered, and in the 12 week follow-up visit the glottis was free of lesions.

Exclusion criteria: the patients with (I) previous head and neck malignancy, (II) history of head and neck irradiation, or (III) the presence of lesions in endoscopic evaluation suggestive of a neoplasm.

The average age of people in the entire sample was 61.3 years (SD = 10.0, Me = 62, range: 26–84), and 19.8% of people were women. 79.2% of people were current or former smokers, 62.3% declared alcohol consumption. In the control group of cases, the average age of 166 people was 60.3 (SD = 10.1, Me = 61, range: 26–81), and 22.3% of people were women. Smoking at some stage in life was declared by 75.3%, and drinking alcohol by 62.7%. In the group containing only recurrent cases, the mean age was 65.3 (SD = 8.1, Me = 67, range: 48–84) and 9.8% were women. Smoking at some stage of life was declared by 95.1% of people, and drinking alcohol by 61.0%.

All patients were assessed by using a trans-nasal flexible video-endoscope (Olympus Medical System Corporation, Tokyo, Japan) using white light (WL), and narrow-band imaging (NBI). In the first step, the VFL in WL according to texture, color, size, redness, symmetry and thickness (according to the Chen 2019 classification^10^) was observed. Afterwards, the NBI view was obtained, and the vascular pattern was assessed by paying close attention to the presence of intrapapillary capillary loops (IPCLs), (according to Ni 2019^11^ and ELS 2015 classifications). For high-risk leukoplakia in the Chen classification, the cut-off point was 3 (elevated and rough leukoplakia), in the Ni classification the cut-off point was 5 (IPCLs outside leukoplakia) and in the ELS classification, the cut-off point was 2 (perpendicular vessels). All details are described in our previous articles^3,7^. All patients underwent TOLM, and the specimens were sent for final histology. Histopathological diagnosis was performed according to the WHO classification system^12^.

The first follow-up visit was scheduled at 12 weeks, subsequently 8 to 12 weeks thereafter for a minimum of 2 years. The basis of the visits was a medical history, voice assessment (not included in this paper), and NBI and WL flexible video-endoscope examination.

The following variables were excluded from the analysis due to insufficient differentiation: “voice work” (1 patient), “bronchial asthma” and “anti-asthmatic drugs” (6 patients).

The primary measure of our study was to investigate whether morphological features of pVFL in WL, vascular pattern in NBI, and primary histological findings could predict VFL recurrence.

The main predictive variables taken into consideration were the vascular pattern, according to the Ni classification (2019), the morphological characteristics, according to the Chen classification (2019), and the histological results, according to WHO (2017) in pVFL, according to the presence of the rVFL. Additional variables were: the patient’s age, sex, smoking history, alcohol history, and location: anterior commissure (AC) involvement, uni- or bilateral lesions, and uni- or multifocal lesions.

Finally, the following independent variables were included in the analysis: age (numerical variable), sex (male *vs.* female), smoking habits (current or past smokers *vs.* never a smoker), alcohol drinking (present *vs.* absent), localization (uni- *vs.* bilateral lesions), AC involvement (1 *vs.* 0), focus (the uni- *vs.* multifocal nature of the lesions), the WLI, by using Chen 2019 classification (categorized as [1] flat and smooth; [2] elevated and smooth; [3] rough), findings in NBI, using Ni 2019 classification (I–VI; coded as “Ni2019” variable) and its binarized version (0, when Ni2019 ≤ III *vs* 1, when Ni2019 > III), and histopathology results, using WHO 2017 (categorized as [0] Low-grade dysplasia; [1] High-grade dysplasia; [2] invasive Ca whereby^2^ values in pVFL were excluded from the study). All data is presented in the Supplementary Table.

### Statistical methodology

Our statistical analysis consisted of four parts. Firstly, we compared the characteristics of leukoplakia before the recurrence (pVFL) and after the recurrence (rVFL). This part was performed exclusively on the rVFL group, which consisted of 41 people. Secondly, we compared the characteristics of the rVFL group (*n* = 41), and the one in which relapse did not occur, treated as the control group (*n* = 166). These two parts were based on descriptive statistics and likelihood ratio tests (*G* tests), which are used to test the independence of two categorical variables and are sometimes classified as tests of greater power than the chi-squared test in the case of smaller samples^13^.

Thirdly, we estimated a logistic regression model to determine significant predictors of leukoplakia recurrence. The modeling process consisted of the following: first a full model was created, considering all potential explanatory variables, and then non-significant variables were excluded from the model one by one to achieve a model that contains only the least potentially significant coefficients, while having the lowest possible AIC and the highest possible pseudo-*R*^*2*^ coefficients. The final model reported in the paper is therefore the reduced model. Such a universal modeling process achieves the best possible model, free of information noise generated by irrelevant variables, while not top-down excluding potentially significant predictors that may not have been detected as strongly significant due to sample size, for example.

Fourthly, we included decision tree creation in the analysis. We treated this stage as providing potentially useful heuristics rules, in addition to logistic regression modeling results. To create a tree which enables statistical inference, we used conditional inference trees (recursive partitioning, but with conditional inference) provided by Hothorn and Zeileis in the package *partykit* for *R* language^14^. Function *ctree* parameters were defined to default, except for the method of distribution of the test statistics computation, which we changed to an often more robust Monte Carlo setting, with 1 000 000 permutations.

The literature highlights that the restrictive treatment of the conventional significance level of 0.05, as an absolute threshold for significant variables, is a misinterpretation of the concept of *p*-value^15,16^. We used, therefore, a more flexible approach in which *p*-values below 0.1 were treated as ‘potentially significant’, and the significance was the subject to gradation (as a continuous measure of compatibility of the data with the assumed model), rather than the erroneous binary focus on the below/above 0.05.

The analysis was performed using the JASP^17^ statistical tool and *R* language (ver. 4.2.2)^18^ with package *partykit*^14^.

## Results

### Differences between before-relapse and after-relapse state in the rVFL group

The complete comparison of the study group (41 patients) divided by the assessment of the pVFL and the assessment of rVFL are available in Table 1, and a description of the comparisons is presented in the following subsections.

**Table 1:** Differences between primary and recurrent leukoplakias in rVFL group.

Variable	Number (percentage)			Likelihood ratio test (*G* test)			
	pVFL (n = 41)	rVFL (n = 41)	Total (n = 82)	*G* value	df	*p*	
Bilateral	11	0	11	16.96	1	< 0.001	***
	(26.8%)	(0%)	(13.4%)				
Multifocal	19	1	20	25.09	1	< 0.001	***
	(46.3%)	(2.4%)	(24.4%)				
Anterior commissure	16	2	18	15.48	1	< 0.001	***
	(39.0%)	(4.9%)	(22.0%)				
WLI = 1	24	16	40	14.22	2	< 0.001	***
	(58.5%)	(39.0%)	(48.8%)				
= 2	5	20	25				
	(12.2%)	(48.8%)	(30.5%)				
= 3	12	5	17				
	(29.3%)	(12.2%)	(20.7%)				
Ni2019 > III	21	27	48	1.82	1	0.178	
	(51.2%)	(65.9%)	(58.5%)				
WHO2017 = 0	26	14	40	16.41	2	< 0.001	***
	(63.4%)	(34.1%)	(48.8%)				
= 1	15	18	33				
	(36.6%)	(43.9%)	(40.2%)				
= 2	0	9	9				
	(0%)	(22.0%)	(11.0%)				

*pVFL* primary vocal fold leukoplakia, *rVLF* recurrent vocal fold leukoplakia.

***p < 0.001.

#### Localization of the VFL recurrences (Figs. 1 and 2)

**Figure 1:**
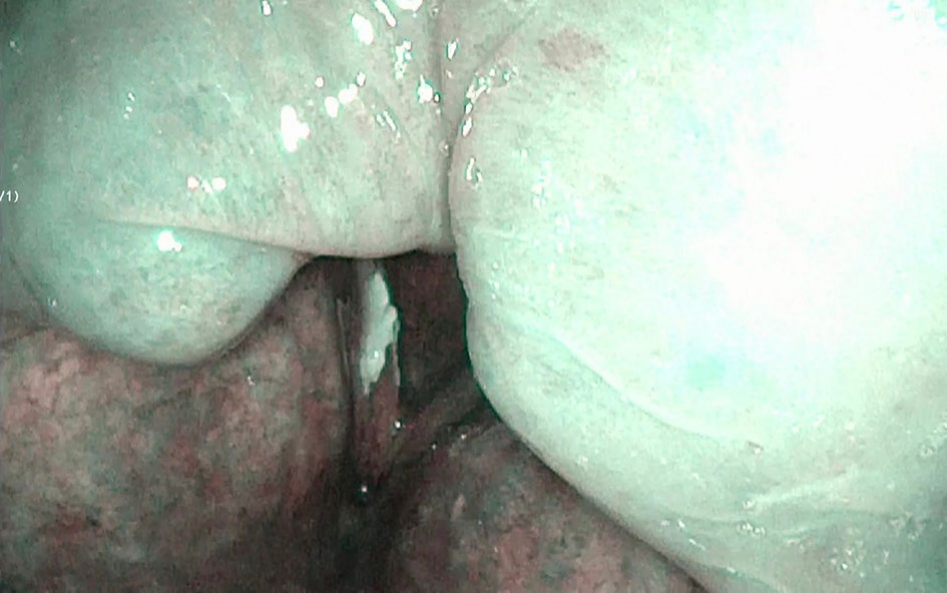
Endoscopic image of the larynx under NBI showing unifocal and unilateral recurrent vocal fold leukoplakia on right vocal fold. In NBI rVFL was determined as type 2 according to Ni 2019 classifiaction. Histopathology from lesions removed from the left vocal fold showed low-risk leukoplakia (WHO 2017).

**Figure 2:**
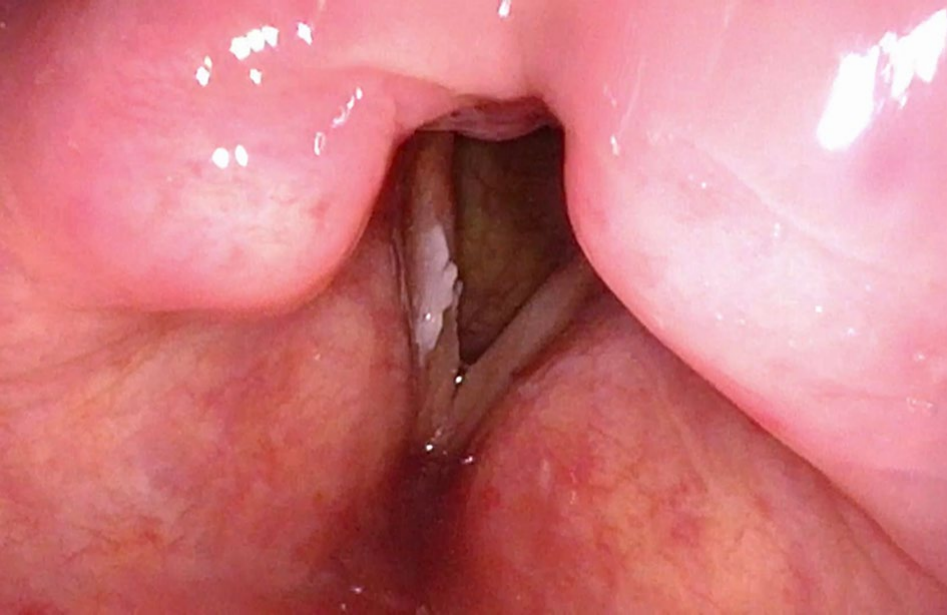
Endoscopic image of the larynx under WLI showing unifocal and unilateral recurrent vocal fold leukoplakia on right vocal fold. In WLI rVFL was determined as type 1 according to Chen 2019 classifiaction. Histopathology from lesions removed from the left vocal fold showed low-risk leukoplakia (WHO 2017).

Taking into consideration the precise localization of the recurrent plaque, 41/41(100%) specimens were unilateral (compared to 30/41 (73.2%) in pVFL; the difference is highly significant: *G*(1) = 17.0, *p* < 0.001), but in 2/41 (4.9%) patients, the lesions spread in the anterior commissure (compared to 16/25 (39.0%) in pVFL, the difference is highly significant: *G*(1) = 15.5, *p* < 0.001). Taking into account the number of rVFL points, 40/41(97.6%) presented unifocal and 1/41(2.4%) multifocal (compared to 19/41 (46.3%) multifocal points among pVFL; the difference is highly significant: *G*(1) = 25.1, *p* < 0.001). There were no relapses involving > 2 locations. Relapses were not multifocal, at least at the stage where patients were identified and referred for treatment.

The binary variable equality of sides” was created for unilateral pVFL, taking the value of 1 when the side of rVFL was the same as pVFL, and 0 when the side of pVFL was different than rVFL. In 26/30 cases (86.7%) leukoplakia recurrence occurred on the same side as pVFL. This percentage is significantly higher than 50% (*t*(29) = 5.81, *p* < 0.001; 95% CI: [73.8%; 99.6%]), indicating a systematic trend.

#### WLI, NBI, and pathological findings in the rVFL group

By using binarized version of Ni 2019 classification: 14/41 (34.1%) patients with rVFL were qualified to low-risk leukoplakia (I-III types) and 27/41 (65.9%) to high-risk leukoplakia (IV-VI types), which is not significantly different comparing to pVFL (21/41 (51.2%) high-risk leukoplakias; *G*(1) = 1.82, *p* = 0.178). According to Chen 2019 classification, 16/41 (39.0%) patients with rVFL had type 1, therefore, belong to low-risk leukoplakia (compared to 24/41 (58.5%) in pVFL), 20/41 (48.8%) patients had type 2 and 5/41 (12.2%) type 3, and both were qualified to high-risk leukoplakia (compared to 5/41 (12.2%) and 12/41 (29.3%), respectively); WLIs structure in rVFL group are significantly different than in pVFL group (G(2) = 14.22, *p* < 0.001).

The histology of the rVFL in 14/41 cases (34.1%) indicated low risk, in 18/41 (43.9%) high-risk leukoplakia, in 9/41 (22.0%) cancer (compared to 26/41 (63.4%), 15/41 (36.6%), and 0 in pVFL group, respectively). The difference between groups is highly significant (*G*(2) = 16.41, *p* < 0.001), but from this alone, one cannot derive a higher cancer risk for rVFL, since the selection criterion for the study was the non-cancerous nature of pVFL, therefore, for pVFL, by definition, the percentage of WLI = 2 is artificially 0.

### Comparison of the rVFL group with the non-recurrence group

Afterward, the patients’ dependent variables and pVFL classification variables were compared between the rVFL group and the patients without recurrence group (control group). The full results of the comparison are presented in Table 2, while a description of the table is included below.

**Table 2:** Differences between patients and pVFL characteristics in recurrence and control group.

Variable	Number (percentage)			Likelihood ratio test (G test)			
	Control group (*n* = 166)	Recurrence group (*n* = 41)	Total (*n* = 207)	*G* value	df	*p*	
Smoking	125	39	164	9.96	1	0.002	**
	(75.3%)	(95.1%)	(79.2%)				
Female	37	4	41	3.70	1	0.054	^○^
	(22.3%)	(9.8%)	(19.8%)				
Alcohol drinking	104	25	129	0.04	1	0.843	
	(62.7%)	(61.0%)	(62.3%)				
Bilateral	28	11	39	1.99	1	0.158	
	(16.9%)	(26.8%)	(18.8%)				
Multifocal	58	19	77	1.79	1	0.180	
	(34.9%)	(46.3%)	(37.2%)				
Anterior commissure	33	16	49	6.14	1	0.013	*
	(19.9%)	(39.0%)	(23.7%)				
WLI = 1	96	24	120	8.69	2	0.013	*
	(57.8%)	(58.5%)	(58.0%)				
= 2	48	5	53				
	(28.9%)	(12.2%)	(25.6%)				
= 3	22	12	34				
	(13.3%)	(29.3%)	(16.4%)				
Ni2019 > III	42	21	63	9.80	1	0.002	**
	(25.3%)	(51.2%)	(30.4%)				
WHO2017 = 1	38	15	53	3.06	1	0.080	^○^
	(22.9%)	(36.6%)	(25.6%)				

^○^*p* < 0.10; *p < 0.05; **p < 0.01.

#### Patients’ dependent factors: age, gender, smoking habits, alcohol drinking

VFL group (41 patients) was characterized by significantly higher mean age (65.3 vs 60.3, *t*(205) = 2.89, *p* = 0.004, *d* = 0.504) and proportion of current or former smokers (95.1% vs. 75.3%, *G*(1) = 9.96, *p* = 0.002), and a potentially significantly higher proportion of men (90.8% *vs* 77.7% in control group, *G*(1) = 3.70, *p* = 0.054), compared to the control group. There was no significant difference in the percentage of people declaring alcohol drinking (61.0% vs. 62.7%, *G*(1) = 0.04, *p* = 0.843).

#### Localisation of pVFL

In patients in the rVFL group, there were no statistically significant differences in the frequency of multifocal (46.3% in the recurrence group vs. 34.9% in the control group, *G*(1) = 1.79, *p* = 0.0180) and bilateral pVFL (26.8% vs. 16.9%, *G*(1) = 1.99, *p* = 0.0158). There was, however, a significant difference in the presence of anterior commissure: such pVFLs were much more frequent in the rVFL group (39.0% vs. 19.9%, *G*(1) = 6.14, *p* = 0.013).

#### Classifications of pVFL

In the recurrence group, type 3 WL assessment (elevated and rough) was significantly more frequent than in the control group, and this increase is close to the type 2 decline (type 3: 29.3% vs. 13.3%, type 2: 12.2% vs. 28.9%, type 1: 58.5% vs. 57.8%, *G*(2) = 8.69, *p* = 0.013). The assessment of Ni 2019 indicated the highly significant dependence between the presence of the rVFL and the assessment of high-risk leukoplakia, i.e. IV-VI type in Ni 2019 (Ni > III in rVFL group: 51.2% vs. 25.3% in the control group, *G*(1) = 9.81, *p* = 0.002). The histology results are slightly different between groups, with more frequent high-risk leukoplakias in the rVFL group, but the difference is only potentially significant (36.6% vs. 22.9% of high-risk WHO2017 assessments, *G*(1) = 3.06, *p* = 0.080).

### VFL recurrence risk model based on multivariate analysis

Following the procedure described in the statistical methodology section, the multivariate logistic regression model was estimated to explain the factors influencing leukoplakia recurrence. The overall model test Pseudo-*R*^2^ measures indicate the very good overall performance of the model (McFadden’s *R*^2^ = 0.182 and Nagelkerke’s *R*^2^ = 0.263; according to McFadden (1977, p. 35), the value of McFadden’s *R*^2^ within 0.2–0.4 range indicates excellent fit of the model). The model has no problems with multicollinearity (each VIF < 1.7).

According to the model, smoking is the factor that significantly and most strongly increases the likelihood of rVFL (odds ratio (OR) [95% CI] = 5.647 [1.241; 25.697], which means that smokers compared to non-smokers have on average about 5.5 times higher odds to develop rVFL; note that an odd is the ratio of the probability of occurrence of a feature to the probability of non-occurrence of a feature, hence it is transformable to the probability of occurrence, but not equal to it (*z* = 2.239, *p* = 0.025). A highly significant coefficient of “age” variable indicates that older people are on average more likely to have rVFL (OR = 1.065 [1.017; 1.115], *z* = 2.653, *p* = 0.008): an age group 10 years older on average is associated with an approximately 1.88 (1.06510) times greater odds of rVFL. The model slightly suggests that women typically have lower odds of rVFL, but this relationship is only potentially significant (OR = 0.359 [0.107; 1.197], *z* = − 1.668, *p* = 0.095).

The model shows that the presence of IPCLs, specifically type IV, V and VI, according to Ni 2019 classification, was associated with a significantly higher risk of the rVFL (OR = 3.407 [1.309; 8.872], *z* = 2.511, *p* = 0.012). WLI = 2 significantly decreases rVFL risk, compared to WLI = 1 (OR = 0.25 [0.074; 0.841], *z* = − 2.239, *p* = 0.025), while WLI = 3 has no effect (*p* = 0.802). The full results of the multivariate logistic regression analysis are presented in Table 3.

**Table 3:** Logistic regression model for recurrence of leukoplakia response variable.

	Estimate	SE	*z*	Wald statistic	*p*		OR [95% CI]	
(Intercept)	− 3.094	0.783	− 3.953	15.626	< 0.001	***	0.045 [0.010; 0.210]	
Smoker	1.731	0.773	2.239	5.014	0.025	*	5.647 [1.241; 25.697]	
Age	0.063	0.024	2.653	7.04	0.008	**	1.065 [1.017; 1.115]	
Female	− 1.026	0.615	− 1.668	2.781	0.1095	^○^	0.359 [0.107; 1.197]	
Ni2019 > III	1.226	0.488	2.511	6.305	0.012	*	3.407 [1.309; 8.872]	
WLI = 2 (ref: 1)	− 1.387	0.62	− 2.239	5.014	0.025	*	0.25 [0.074; 0.841]	
= 3 (ref: 1)	0.145	0.577	0.251	0.063	0.802		1.156 [0.373; 3.583]	

Model performance measures	Deviance	df	AIC	*Χ*^2^	*p*		*R*^*2*^_McFadden_	
Null model	206.05	206	208.05					
Current model	168.60	200	182.60	37.45	< 0.001	***	0.182	0.263

*Note* age variable was centered by subtracting its mean to obtain interpretable intercept.

^○^*p* < 0.10; *p < 0.05; **p < 0.01; ***p < 0.001.

### Heuristics model of VFL recurrence risk based on conditional inference tree modeling

**Figure 3:**
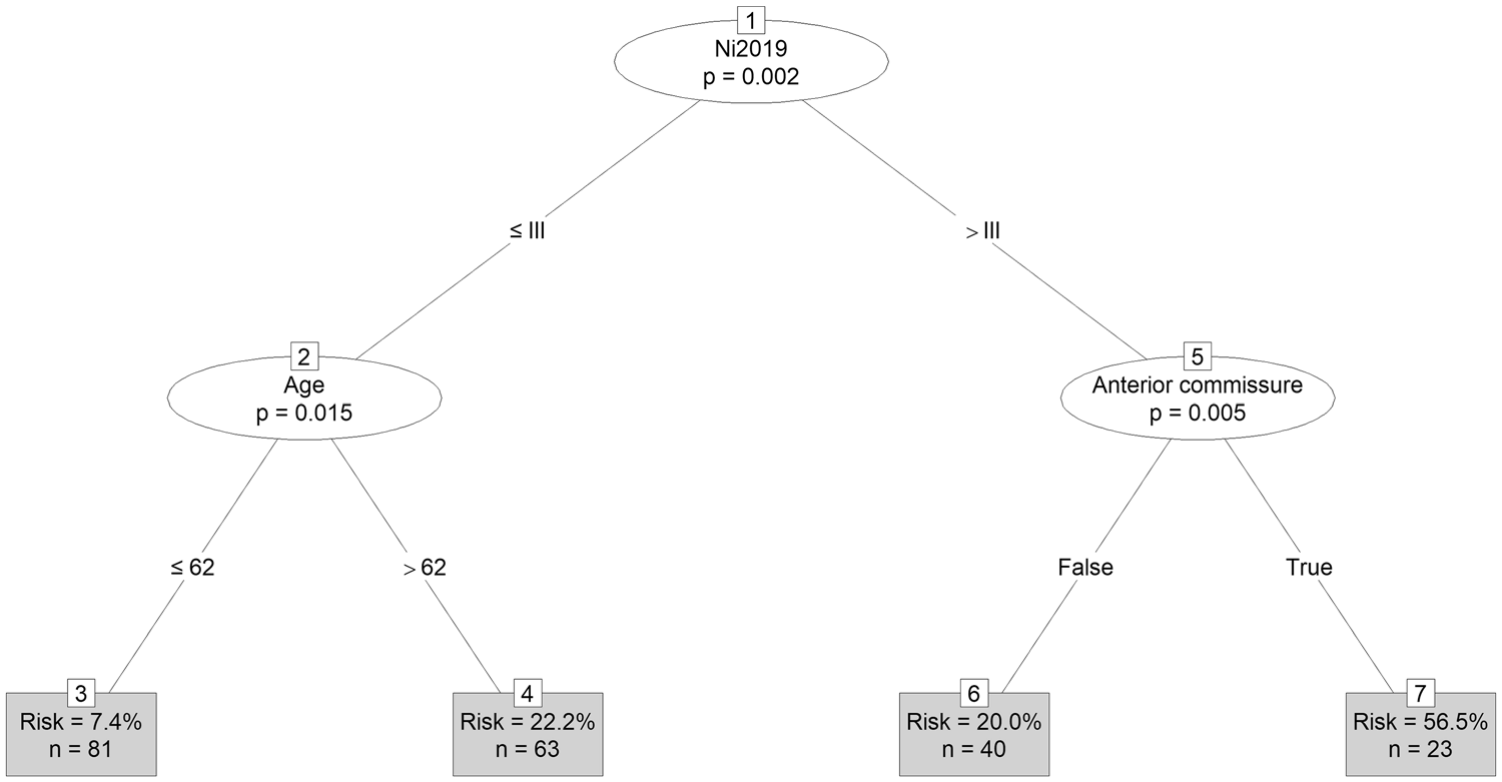
Visualization of the conditional inference decision tree, with recurrence of the VFL as the response variable; "p" represents *p*-value of independence test against variable listed inside an ellipse; "Risk" represents percentage of patients having recurrent VFL in the group; "n" represents a number of patients in the group.

To obtain a simple and easily interpretable model of risk, supplementary to logistic regression analysis, we created a conditional inference decision tree. The created tree is presented in Fig. 3. The information in the tree is given as follows: splits use a logical condition, which if it is met, we go along this path. Each path leads to a smaller group (at the next sublevel of the tree). The final vertexes of the tree display three pieces of information: the category of the percentage of Recurrence = 1 (patients from the rVFL group) in that vertex, the number of all cases that fall into that cluster, and the error of the model.

The tree suggests that the most important variable distinguishing groups in terms of leukoplakia recurrence is the binarized version of the Ni2019 scale (0: I, II, III categories; 1: IV, V, VI categories): Ni2019 > III is combined with significantly higher risk of rVFL (*p* = 0.002). In the Ni2019 > III group, the next strongest differentiating variable was localization in AC (*p* = 0.005). When it took a value of 1 in the group that had higher Ni2019 values, the probability of rVFL increased from about 20% to about 57%. Finally, age is an important variable in the group with lower Ni2019 values (*p* = 0.015): younger people (aged 62 or less), when their pVFL was Ni2019 category III or lower, had only an approximate 7% probability of rVFL, while older already had a probability of about 22%. It is worth mentioning that if Ni2019 was excluded from the analysis, the most significant variable (dividing the set as the first) turned out to be smoking (*p* = 0.004): smokers were about five times more likely to have rVFL than others. The results provided by the tree mostly coincide with the logistic regression model, but uncovered an additional, potentially diagnostically relevant variable: presence in the anterior commissure.

## Discussion

Plenty of research is devoted to the characteristics of VFL based on which the risk of malignant transformation can be predicted, while risk factors for VFL recurrence have still not been cleared. Bearing in mind that in VFL the balance between intensive treatment and the voice result remains the most important issue, each subsequent procedure can be highly detrimental to the quality of voice. Thus, the aim of this paper was to do a detailed analysis of the risk factors for VFL recurrence despite adequate primary treatment, and to define the combination of factors that play the main role in resolving the divide in the approach to the first treatment.

The natural history of rVFL is not well-established^6,19,20^. Even a complete resection of the pVFL does not eliminate recurrences. Our results are consistent with a few previous results, presented by Park et al. and Lee et al., showing that rVFL is not rare (17.6% and 19%, respectively)^21,22^, compared to 19.8% of patients in the current study. Due to the challenge of predicting the appearance of the rVFL, we wanted to specifically explore NBI (Ni 2019)^11^ and WLI (Chen 2019^10^) scores, and additionally find other relevant risk factors.

WL endoscopy is the basic method in glottis assessment; however, it is an insufficient diagnostic tool to predict leukoplakia recurrence which has so far been made based on WL scales. The classification by Chen et al. 2019^10^, dedicated to VFL, focuses on the morphological features of the lesion and adds new important information about it. In this classification, 3 VFL types have been isolated: type 1 – present flat and smooth, type 2 – elevated and smooth, and type 3 – rough leukoplakia, well-correlated with pathological grades. Our results, according to WL (Chen 2019), were potentially counterintuitive because of the decreasing risk for WL = 2, and no differences between 3 and 1 categories of WL rely on the fact of controlling Ni2019 variable: the exploratory analysis revealed that WL = 3 and Ni2019 > III carry similar information, making the impact of WL = 3 disappear after including both of these variables in the model. If a similar model was built, but without the Ni2019 variable, the coefficient at WL = 2 vs WL = 1 would become insignificant (*p* = 0.134), and the coefficient at WL = 3 vs WL = 1 would be significant (*p* = 0.042) and positive (OR 2.595 [1.037; 6.495]), signaling an increased risk of rVFL for this category of WLI. This comparison suggests, however, a higher information value of Ni2019 than WL, in the prediction of leukoplakia recurrence.

NBI has been established as a very useful tool in the assessment of microvascular morphology of mucosal surfaces. In recent years, some authors proved the important role of NBI in vascular assessment of the VFL in prediction of malignant transformation^23,24^. The significance of combining (cross-checking) two classifications, Ni 2019^11^ and ELS 2015^25^, and comparing findings from both with the final histology results was proved^3,7^. In our study, the correlation between the NBI vascular pattern of the pVFL and the recurrence was examined and a conclusion has been drawn—patients with high-risk pVFL, who scored Ni2019 type IV, V and VI, had a higher risk of developing the rVFL and require closer follow-up. This finding is robust, since the variable was included in the logistic regression model, as well as the decision tree, in both cases as a highly significant predictor. This finding may contribute to recommending the extension of the VFL diagnosis by the corresponding classification grade on the discharge charts after the first treatment, which will clarify the nature of the lesion.

Currently, there is no consensus about the role of pathological grade pVFL on the risk of recurrence for leukoplakia. Some authors believe that the histological grade of VFL plays an important role^26,27^; however, some report the contrary^28,29^. In our paper, the results showed more frequent high-risk leukoplakias in the rVFL group, but the difference is only potentially significant and only in univariate analysis (36.6% vs. 22.9% of high-risk WHO2017 assessments, *G*(1) = 3.06, *p* = 0.080; the variable was not included in the regression model, nor in the decision tree model). This suggests that even if WHO2017 has a significant effect, it has less informative value than other variables controlled in the multivariate analysis.

Patient-related factors are discussed with particular emphasis because they happen to have a significant predictive impact on VFL recurrence. Jabarin et al.^30^ showed an increased malignant transformation rate in rVFL cases, especially in male and heavy smokers, and recommended close follow-up of this subgroup, although the authors did not analyze the probability of recurrences. Our study is consistent with Jabarin et al. findings. Firstly, smoking has been included as a significant predictor in the logistic regression model; its OR was the highest among all of the predictors. Moreover, in the decision tree model created in our study, after the exclusion of the Ni 2019 factor from the analysis, the most significant variable turned out to be smoking (*p* = 0.004): smokers were about five times more likely to have rVFL than others. Secondly, our study showed that women typically have lower odds of rVFL, but this relationship is only potentially significant (OR = 0.359 [0.107; 1.197], *z* = − 1.668, *p* = 0.095), which probably is an effect of a small number of women in the sample (41), not enough to detect smaller effects.

To summarize, our research showed twofold results. Our model of risk in the form of a decision tree showed the three most important variables in the assessment of rVFL presence: the pVFL determined as Ni2019 > III, localization of the pVFL in the anterior commissure, and age > 62. However, our risk model based on logistic regression analysis proved that smoking is the main factor that is combined with a high risk of recurrence. Similarly to the decision tree, our regression model showed that Ni2019 > III and age are also important variables, with higher values increasing recurrence risk. Therefore, more aggressive primary treatment and close follow-up could be advised in patients with these factors. On the other hand, the rVFL is much less affected by the first treatment than the patient-dependent factors. The combination of age and addictions plays a decisive role. Therefore, it seems that in the selected group of patients, psychological intervention in the initial stages would play a decisive role in abandoning the harmful lifestyle.

The strength of this study was the unique presentation of the large primary VFL group with long follow-up data, focused on rVFL occurrence, and elucidating the risk factors of VFL recurrence. The innovative method brings together the patient-related variables, and VFL assessment both in NBI and WLI classifications, according to Ni 2019^11^ and Chen^10^. The first to our knowledge VFL Recurrence Risk Model was created with practical implications.

The limitation of this study was that the primary procedures were performed by different surgeons. Moreover, the two-year postoperative follow-up period considered in the study may be regarded as short. Finally, the estimation of the effects affecting the leukoplakia recurrence would benefit if the recurrence group, i.e., the number of recurrent lesions, will be larger than in the current study. The voice outcome in rVFL treatment is a complementary but crucial element not included in this paper and should be a challenge for future research.

## Conclusions

The ability to detect the potential of primary VFL to recur and patient categorization for more aggressive primary treatment remains the diagnostic challenge. The algorithm combining patients’ dependent variables, white and blue light, and the combination of two classifications improve the specificity and the positive predictive value of the presented VFL Recurrence Risk Model.

Future studies on a larger group of patients and controlling for additional variables, such as the presence of gastro-oesophageal reflux among patients, are desirable. Also, further studies addressing the associations of recurrent leukoplakia and multiple surgical interventions on voice outcome, taking into account indicators such as VHI or GRBAS, will be valuable.

### Supplementary Information

Supplementary Information.

## Data Availability

The datasets generated during and/or analyzed during the current study are available from the corresponding author on reasonable request.
